# Patterns of Imaging Requests By General Practitioners for People With Musculoskeletal Complaints: An Analysis From a Primary Care Database

**DOI:** 10.1002/acr.25189

**Published:** 2023-09-27

**Authors:** Romi Haas, Alexandra Gorelik, Denise A. O'Connor, Christopher Pearce, Danielle Mazza, Rachelle Buchbinder

**Affiliations:** ^1^ Monash University Melbourne Victoria Australia; ^2^ Outcome Health Blackburn Victoria Australia

## Abstract

**Objective:**

The aim of this study was to examine imaging requested by general practitioners (GPs) for patients with low back, neck, shoulder, and knee complaints over 5 years (2014–2018).

**Methods:**

This analysis from the Australian Population Level Analysis and Reporting database included patients presenting with a diagnosis of low back, neck, shoulder, and/or knee complaints. Eligible imaging requests included low back and neck x‐ray, computed tomography (CT), and magnetic resonance imaging (MRI); knee x‐ray, CT, MRI, and ultrasound; and shoulder x‐ray, MRI, and ultrasound. We determined number of imaging requests and examined their timing, associated factors, and trends over time. Primary analysis included imaging requests from 2 weeks before diagnosis to 1 year after diagnosis.

**Results:**

There were 133,279 patients (57% low back, 25% knee, 20% shoulder, and 11% neck complaints). Imaging was most common among those with a shoulder (49%) complaint, followed by knee (43%), neck (34%), and low back complaints (26%). Most requests occurred simultaneously with the diagnosis. Imaging modality varied by body region and, to a lesser extent, by gender, socioeconomic status, and primary health network. For low back, there was a 1.3% (95% confidence interval [95% CI] 1.0–1.6) annual increase in proportion of MRI and a concomitant 1.3% (95% CI 0.8–1.8) decrease in CT requests. For neck, there was a 3.0% (95% CI 2.1–3.9) annual increase in proportion of MRI and a concomitant 3.1% (95% CI 2.2–4.0) decrease in x‐ray requests.

**Conclusion:**

GPs commonly request early diagnostic imaging for musculoskeletal complaints at odds with recommended practice. We observed a trend towards more complex imaging for neck and back complaints.

## INTRODUCTION

Diagnostic imaging for the majority of people with nonspecific regional musculoskeletal complaints has limited value. First, degenerative findings are common in asymptomatic people and increase with age. For example, a systematic review including 33 studies (n = 3,110) found vertebral disc degeneration is present in approximately 37% and 96% of asymptomatic 20‐ and 80‐year‐olds, respectively (1). Similarly, other reviews have found knee osteophytes (2) and rotator cuff abnormalities (3) are seen in imaging of asymptomatic people, more commonly in those with increasing age. Second, because imaging abnormalities are so common in asymptomatic people, their clinical relevance in symptomatic individuals is questionable. For example, two recent longitudinal studies, one based on lumbar spine x‐ray (4) and the other on MRI (5), reported no association between degenerative findings and current or future back pain. Finally, evidence suggests patient‐reported outcomes such as pain and function do not improve in patients who receive imaging compared with those who do not (6, 7).

**SIGNIFICANCE & INNOVATIONS**

- This study presents data from a large sample of people with regional musculoskeletal complaints that are broadly representative of the wider population using routinely collected data.
- We observed general practitioners commonly request early diagnostic imaging for musculoskeletal complaints and a trend toward more complex imaging for neck and low back complaints.
- These findings are at odds with recommended practice.
- Multifaceted strategies to improve appropriate imaging requests for people with musculoskeletal complaints are urgently needed.

For these reasons, clinical care standards and clinical practice guidelines discourage imaging for regional musculoskeletal complaints unless serious pathology is suspected, there is an unsatisfactory response to conservative care, and/or imaging is likely to change management (8, 9, 10). In addition, Choosing Wisely recommendations such as “do not undertake imaging for low back pain for patients without indications of an underlying serious condition” and “do not request shoulder ultrasound to diagnose nonspecific shoulder pain which on clinical evaluation is suggestive of rotator cuff pathology and in which surgery is not planned” (11) have been developed as part of a global campaign encouraging conversations about reducing unnecessary tests, treatments, and procedures.

Despite these recommendations, there is evidence that imaging rates for musculoskeletal complaints have increased over time. For example, a systematic review including 27 studies found a 53.5% increase in complex imaging requested for low back pain over 21 years from 1995–2015 (12). In Australia, the likelihood of general practitioner (GP)–requested imaging tests increased by 9% from one period between 2002 and 2005 to a second period between 2009 and 2012 for patients with shoulder and knee complaints (13). Similarly, the likelihood of GP‐requested CT scans for patients with neck complaints more than doubled from 2.6%–5.9% during the same time periods (13).

Monitoring imaging trends over time is important to ensure the provision of high‐quality care. However, in Australia, previous studies seeking to understand imaging requests in primary care have relied on data from the Medicare Benefits Schedule (14) or cross‐sectional databases such as the Bettering the Evaluation and Care of Health dataset (13), which cannot evaluate changes in GP imaging requests over time for people with specific conditions.

General practice databases using routinely collected data from electronic medical records (EMRs) provide an efficient way of examining imaging requests over time. To our knowledge, these databases have not yet been used to examine patterns of care over time for people with musculoskeletal complaints. The objective of this study was to investigate the imaging requested by GPs for people with low back, neck, shoulder, and knee complaints using prospectively collected longitudinal data among general practices participating in the Population Level Analysis and Reporting (POLAR) database. It specifically examined the proportion of patients with imaging requests, imaging modalities, timing of diagnostic imaging requests, associated factors, and changes in diagnostic imaging requests over a 5‐year study period. This study forms part of a larger project that has examined patterns of care for people with musculoskeletal complaints provided also including GP consultations, referrals to other health care providers, and prescriptions for pain relief (15).

## MATERIALS AND METHODS

### Data source and setting

This is a retrospective longitudinal analysis of deidentified data from the POLAR database. This database extracts patient‐related information from every GP/patient encounter directly from the EMRs of consenting general practices (n = 301) within the Primary Health Networks (PHNs) of Eastern Melbourne, South‐Eastern Melbourne, and Gippsland within Victoria, Australia. After excluding practices with inconsistent activity recording during the study period, 269 practices were included in the data analysis. The study protocol, including a detailed description of the rationale, aims, and methods (including data cleaning and sample size consideration), has been previously published (15). We conducted the study following the Reporting of studies Conducted using Observational Routinely‐collected Data guidelines (16).

### Participants

We included patients with at least one GP face‐to‐face consultation and a diagnosis of an eligible atraumatic low back (≥18 years), and/or neck, shoulder, or knee complaint (≥45 years) between January 1, 2014, and December 31, 2018. These age restrictions were chosen because the prevalence of most musculoskeletal conditions increases markedly after the age of 45 years, except for low back pain, which increases after the age of 18 years (17). We also excluded traumatic injuries because these are not likely to be primarily managed by a GP, imaging may be warranted, and these injuries are more common in people aged 18–44 years (18).

### Data extracted

For this analysis, we extracted patient characteristics, dates, and diagnoses of eligible musculoskeletal complaints and dates and modalities of eligible imaging requests, which included low back and neck x‐ray, CT, and MRI; knee x‐ray, CT, MRI, and ultrasound; and shoulder x‐ray, MRI, and ultrasound. Shoulder CT was excluded because it is rarely requested by GPs for atraumatic shoulder pain in our setting. We separated diagnostic imaging from image‐guided procedural requests (ie, intraarticular or soft‐tissue injections and hydrodilatation). Compared with publicly available Medicare statistics, our dataset captured all GP imaging requests regardless of their funding source. This included those eligible for a Medicare rebate paid by the Australian government and those that were fully patient funded.

The POLAR database extracts structured data from various fields of the EMR, deidentifies it, and uses a combination of automated and manual processing to code the data so that they can be used for research purposes. Eligible musculoskeletal complaints were derived from diagnoses that are mapped and coded to Systemized Nomenclature of Medicine Clinical Terms within POLAR. A list of eligible diagnostic codes was reported in our protocol (15) and is available from https://clinicalcodes.rss.mhs.man.ac.uk/medcodes/article/174/. Our research team used an inductive coding process to select and categorize eligible imaging records. Our coding accounted for over 95% of the 845,400 diagnostic and procedural imaging records identified (15).

### Data analysis

All relevant data were extracted from the POLAR Structured Query Language database and exported into Stata version 15 (StataCorp LP) for data management and analysis. For this analysis, we included imaging requested for an individual patient from 2 weeks prior to the date of the first eligible musculoskeletal diagnosis until 1 year following diagnosis or for patients with an eligible musculoskeletal complaint diagnosed in 2018 until the end of 2018. We included the 2 weeks before the date of diagnosis because imaging often precedes diagnosis (19). In addition, a preliminary analysis showed the majority of images requested in the 6 months before diagnosis occurred in the 2 weeks before (Supplementary Figure 1). We also conducted a sensitivity analysis including imaging requested during the entire follow‐up period (until December 31, 2018).

We determined the number (percentage) of patients with at least one eligible diagnostic imaging request (overall and for each modality), number (percentage) and modality of diagnostic imaging requests, and number (percentage) of image‐guided procedure requests by body region. Requested imaging for multiple modalities or procedures were counted separately. For the timing, regression, and trend analyses, we only included diagnostic imaging requests. The median (interquartile range [IQR]) time (days) from index diagnosis until the first diagnostic imaging request for each body region was also determined.

Multivariable logistic regression was used to examine the association between diagnostic imaging modality requested (x‐ray, CT, MRI, and ultrasound) and patient‐ and practice‐related characteristics including gender, socioeconomic status (lowest quintile or other), residential remoteness (metropolitan or other), practice PHN (Eastern Melbourne, South‐Eastern Melbourne, or Gippsland), and body region affected (low back, neck, shoulder, or knee).

We reported odds ratios (ORs) with a 95% confidence interval (95% CI) adjusted for age and time since diagnosis. *P* values less than 0.01 were interpreted as statistically significant to account for multiplicity, and a change in OR of ≥10% was interpreted as clinically relevant. In the absence of published data about what would be clinically important, we determined this a priori based upon our clinical judgement. Gippsland was chosen as the reference PHN because this is a predominantly regional and remote area compared with Eastern Melbourne and South‐Eastern Melbourne PHNs, which are predominantly metropolitan (20). Knee was chosen as the body region reference because this was the only site that included all imaging modalities, enabling reporting of the odds of receiving an imaging request of a specific modality for a low back, neck, or shoulder complaint compared with a knee complaint.

Trend analysis was used to examine the longitudinal changes in the proportion of patients with imaging requested and the proportion of imaging requests for each modality and body region between 2014 and 2018. *P* values less than 0.05 were interpreted as statistically significant, and a change in either direction of 1% or more per year was considered clinically relevant also determined a priori based upon clinical judgement. Based on the results of a recent trial that evaluated the effect of audit and feedback for reducing musculoskeletal imaging, a 1% reduction in imaging rate would result in approximately 4,700 fewer imaging requests per year (21). Based upon an estimated average of 1.5 imaging requests per person and assuming an imaged proportion of 25% within our cohort (12), this would translate into a change of 10% or more in the OR.

### Ethics approval and consent to participate

This study was approved by the Cabrini Human Research Ethics Committee and Monash University Human Research Ethics Committee (reference numbers 02‐21‐01‐19 and 16975, respectively) and was conducted in accordance with the Declaration of Helsinki. We did not obtain participant consent because all data were anonymized. Outcome Health holds a standing ethics approval for its collection and custodianship of the deidentified data from the Royal Australian College of General Practice. Outcome Health and the individual PHNs granted permission to access the data used in this study.

## RESULTS

### Study cohort

Our eligible study cohort (133,279 patients, 4,538 GPs, and 269 general practices) has been described previously (22). More than half the cohort were female (n = 73,428, 55%), and approximately two thirds had at least one comorbidity (n = 83,816, 63%). Mean (SD) age of the study cohort at diagnosis was 49.2 (18.5) years for those with low back complaints and 61.9 (12.0), 62.8 (11.8), and 64.2 (11.5) years for those with neck, shoulder, and knee complaints, respectively. Based on diagnostic codes, more than half (n = 76,504, 57%) had a low back complaint, a quarter (n = 33,438) had a knee complaint, a fifth (n = 26,335) had a shoulder complaint, and 11% (n = 14,492) had a neck complaint. This includes one tenth (n = 15,176, 11%) of the cohort who had multiple body regions affected by an eligible musculoskeletal complaint.

### Imaging requests

Over one‐third (n = 49,174, 37%) of the cohort had at least one eligible imaging request (diagnostic or procedural) within the eligible study period. There were 76,249 imaging requests overall, with a median (IQR) of 1 (1, 2) requests per patient.

Imaging requests varied by musculoskeletal complaint. From the 2 weeks before the index diagnosis to 1 year after diagnosis, patients with a shoulder complaint had diagnostic imaging requested most commonly (n = 12,959, 49% patients), followed by knee (n = 14,405, 43%), neck (n = 4,871, 34%), and low back complaints (n = 19,545, 26%) (Table 1). Ultrasound (n = 12,329, 57% requests) and x‐ray (n = 8,718, 40%) were the most frequently requested modality for shoulder complaints, x‐rays for knee complaints (n = 13,879, 62%), CT for low back complaints (n = 11,160, 50%), and MRI (n = 2,064, 37%) and x‐ray (n = 1,960, 36%) were most frequently requested for neck complaints. Over 1 in 10 patients with a shoulder complaint had at least one image‐guided procedure requested compared with less than 1% of patients with low back, neck, and knee complaints. There were 500 requests for shoulder hydrodilatation among 445 (2%) patients with a shoulder complaint.

**Table 1:** Number (%) of diagnostic and procedural imaging requests and number of patients (%) with imaging requests by modality and body region within 2 weeks before to 1 year after index diagnosis*

	Total study cohort (133,279 patients) n (%)	Low back (76,504 patients) n (%)	Neck (14,492 patients) n (%)	Shoulder (26,335 patients) n (%)	Knee (33,438 patients) n (%)
Patients with diagnostic imaging requests					
At least one request	48,253 (36.2)	19,545 (25.5)	4,871 (33.6)	12,959 (49.2)	14,405 (43.1)
X‐ray	26,232 (19.7)	7,485 (9.8)	1,923 (13.3)	7,787 (29.6)	10,258 (30.7)
CT scan	12,379 (9.3)	10,854 (14.2)	1,464 (10.1)	N/A	160 (0.5)
MRI scan	11,031 (8.3)	3,140 (4.1)	2,012 (13.9)	719 (2.7)	5,332 (15.9)
Ultrasound	13,156 (9.9)	N/A	N/A	10,898 (41.4)	2,440 (7.3)
Requests for diagnostic imaging					
Total	71,865 (100)	22,153 (30.8)†	5,513 (7.7)†	21,812 (30.3)†	22,387 (31.2)†
X‐ray	32,313 (45.0)	7,763 (35.0)	1,960 (35.6)	8,718 (40.0)	13,872 (62.0)
CT scan	12,821 (17.8)	11,160 (50.4)	1,489 (27.0)	N/A	172 (0.8)
MRI scan	11,737 (16.3)	3,230 (14.6)	2,064 (37.4)	765 (3.5)	5,678 (25.4)
Ultrasound	14,994 (20.9)	N/A	N/A	12,329 (56.5)	2,665 (11.9)
Patients with procedural imaging request‡					
At least one request	3,731 (2.8)	284 (0.4)	52 (0.4)	3,227 (12.3)	181 (0.5)
Image‐guided injection	3,370 (2.5)	284 (0.4)	52 (0.4)	2,866 (10.9)	181 (0.5)
Hydrodilatation	445 (0.3)	N/A	N/A	445 (1.7)	N/A
Requests for procedural imaging‡					
Total	4,384 (100)	327 (7.5)†	61 (1.4)†	3,793 (86.5)†	203 (4.6)†
Image‐guided injection	3,884 (88.6)	327 (100)	61 (100)	3,293 (86.8)	203 (100)
Hydrodilatation	500 (11.4)	N/A	N/A	500 (13.2)	N/A

* The number of participants with a musculoskeletal condition affecting each body region sums to more than 133,279, because n = 15,176 participants were diagnosed with musculoskeletal symptoms affecting multiple body regions. For proportion of total radiology requests, each patient may have had requests for multiple images and/or modalities for the same body region. CT = computed tomography; MRI = magnetic resonance imaging; N/A = not applicable.

† Percentage of imaging requests was calculated from the total study cohort (n = 71,865 diagnostic and n = 4,384 procedural).

‡ Image‐guided injections were intra‐articular or bursal injection of glucocorticoid, or it was not specified or hydrodilatation (arthrographic distension with glucocorticoid and saline, or it was not specified).

**Figure 1:**
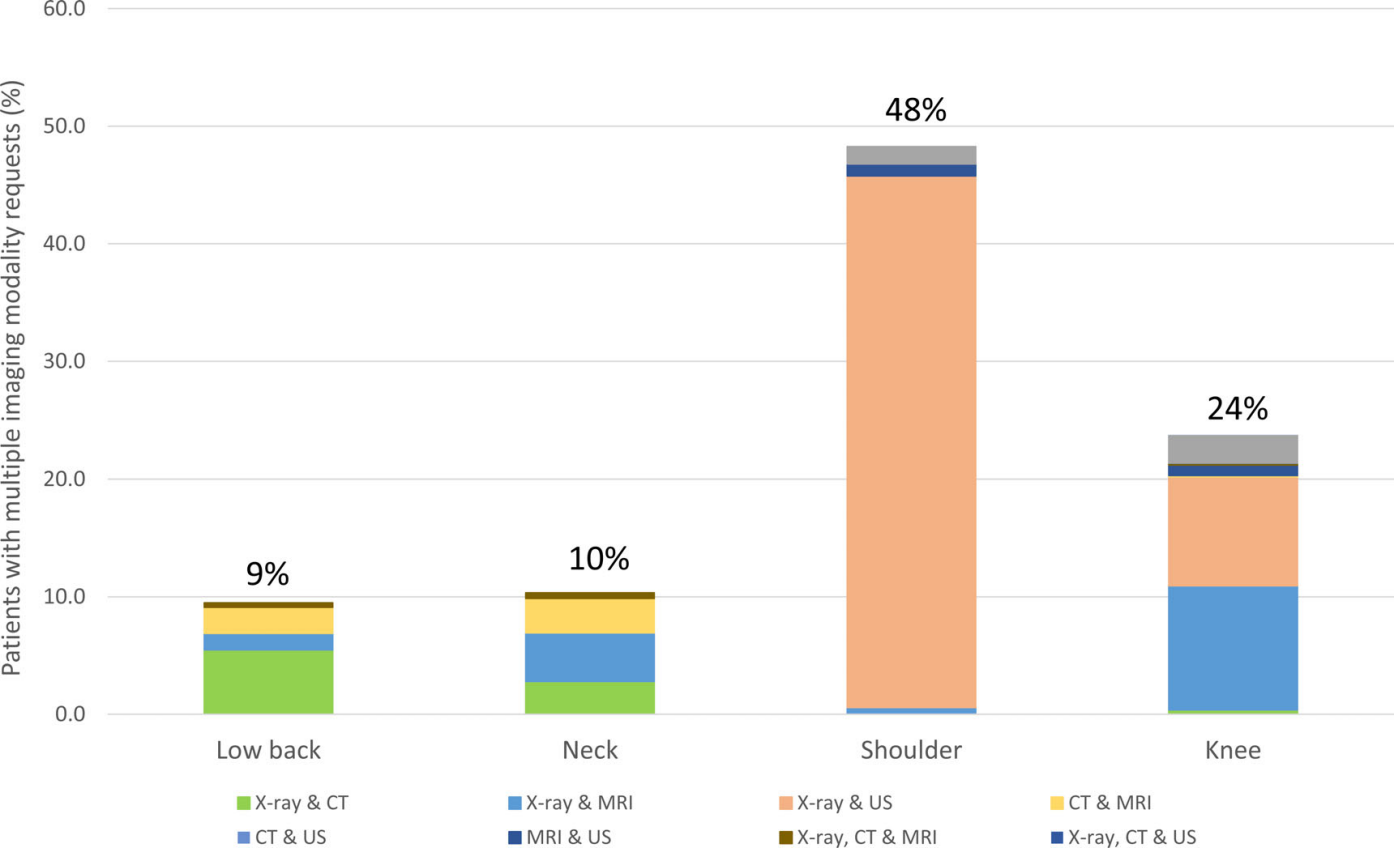
Proportion of patients with multiple diagnostic imaging requests by modality and region. CT = computed tomography; MRI = magnetic resonance imaging; US = ultrasound.

Many patients had requests for more than one type of diagnostic imaging (n = 6254, 48% patients with at least one shoulder imaging request; n = 3419, 24% for knee; n = 1856, 9% for low back; and n = 504, 10% for neck complaints). The most frequent combinations were shoulder x‐ray and ultrasound (n = 5,856, 45% patients with at least one imaging request), knee x‐ray and either MRI (n = 1,521, 11%) or ultrasound (n = 1,333, 9%), and low back x‐ray and CT (n = 1,070, 5%) (Figure 1).

### Timing of diagnostic imaging requests

**Figure 2:**
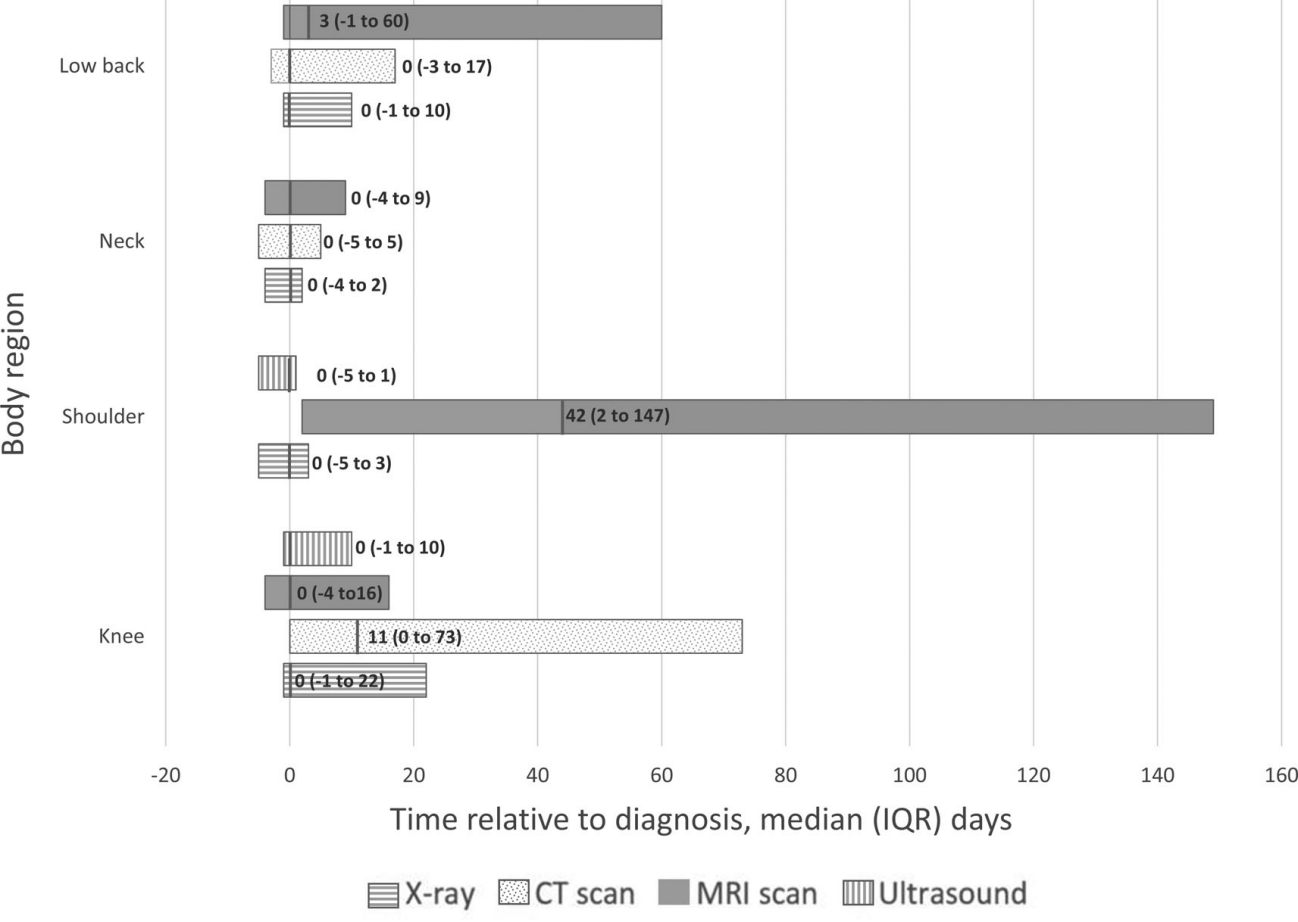
Timing of first diagnostic imaging request by body region and imaging modality, median (interquartile range [IQR]) days since diagnosis. CT = computed tomography; MRI = magnetic resonance imaging.

Almost a third (n = 22,571, 30%) of eligible imaging requests were made within the 2‐week period before diagnosis (Supplementary Figure 1). The median timing was on the same day as the diagnosis except for shoulder MRI, knee CT, and low back MRI, which were all requested at a later time (median [IQR] days: 42 [2–147], 11 [0–73], and 3 [−1 to 60]) (Figure 2).

### Association between diagnostic imaging and patient‐ and practice‐related characteristics

Body region was the strongest predictor of diagnostic imaging requests (Table 2). Compared with patients with knee complaints, the odds of receiving any imaging request were 49% (OR 0.51, 95% CI 0.49–0.52) and 31% (OR 0.69, 95% CI 0.66–0.73) lower for patients with low back and neck complaints, respectively, and 56% higher (OR 1.56, 95% CI 1.50–1.62) for those with shoulder complaints. Compared with patients with knee complaints, those with shoulder complaints were 86% less likely (OR 0.14, 95% CI 0.13–0.15) to receive an x‐ray request than those with knee complaints but were 11 times more likely (OR 11.33, 95% CI 10.23–12.56) to receive an ultrasound request.

**Table 2:** Associations between imaging types and body region affected, patient variables, and GP practice*

	Imaging vs. no imaging, adjusted OR (95% CI)	X‐ray only, adjusted OR (95% CI)	CT scan only, adjusted OR (95% CI)	MRI scan only, adjusted OR (95% CI)	Ultrasound only, adjusted OR (95% CI)
Body region affected					
Knee	1	1	N/A^a^	1	1
Low back	0.51 (0.49–0.52)	0.50 (0.48–0.53)	1	0.37 (0.35–0.40)	N/A
Neck	0.69 (0.66–0.73)	0.49 (0.45–0.53)	0.33 (0.30–0.36)	1.82 (1.66–1.98)	N/A
Shoulder	1.56 (1.50–1.62)	0.14 (0.13–0.15)	N/A	0.08 (0.07–0.10)	11.33 (10.23–12.56)
Patient‐related variables					
Male	1.01 (0.98–1.03)	0.83 (0.80–0.87)	1.21 (1.15–1.28)	1.16 (1.10–1.23)	1.0 (0.93–1.09)
Lowest socioeconomic quintile	0.83 (0.79–0.86)	1.18 (1.10–1.27)	1.17 (1.08–1.28)	0.54 (0.48–0.90)	1.23 (1.07–1.43)
Metropolitan residential location	0.99 (0.94–1.04)	1.01 (0.92**–**1.12)	0.94 (0.83–1.07)	0.94 (0.83–1.07)	0.98 (0.83–1.17)
GP practice					
Gippsland	1	1	1	1	1
Eastern Melbourne	0.92 (0.86–0.98)	0.63 (0.56–0.71)	1.16 (0.99–1.36)	1.69 (1.42–2.01)	2.54 (1.98–3.26)
South‐Eastern Melbourne	0.97 (0.91–1.04)	0.63 (0.56–0.72)	1.26 (1.08–1.47)	1.66 (1.39–1.97)	2.44 (1.90–3.14)

* Bold indicates statistically signficant (*P* < 0.01). All regression models are adjusted for age and time since index diagnosis. They include participants with a single body region affected by a musculoskeletal complaint. *P* < 0.01 was statistically significant. 95% CI = 95% confidence interval; CT = computed tomography; GP = general practitioner; MRI = magnetic resonance imaging; N/A = not applicable; OR = odds ratio.

^a^ There were too few knee CTs to compare (report neck relative to low back).

Irrespective of the musculoskeletal complaint, men were 17% less likely (OR 0.83, 95% CI 0.80–0.87) to receive an x‐ray request and 21% more likely to receive requests for CT (OR 1.21, 95% CI 1.15–1.28) and MRI (OR 1.16, 95% CI 1.10–1.23). Patients living in an area of low socioeconomic advantage were more likely to receive a request for an x‐ray (OR 1.18, 95% CI 1.10–1.27), CT (OR 1.17, 95% CI 1.08–1.28), and ultrasound (OR 1.23, 95% CI 1.07–1.43) but were less likely to receive an MRI request (OR 0.54, 95% CI 0.48–0.90).

Compared with those attending a Gippsland PHN practice, patients attending predominantly metropolitan practices were less likely (Eastern Melbourne [OR 0.63, 95% CI 0.56–0.71] and South‐Eastern Melbourne PHNs [OR 0.63, 95% CI 0.56–0.72]) to receive an x‐ray request but were more likely to receive requests for ultrasounds (Eastern Melbourne [OR 2.54, 95% CI 1.98–3.26] and South‐Eastern Melbourne [OR 2.44, 95% CI 1.90–3.14]) and MRI scans (Eastern Melbourne [OR 1.69, 95% CI 1.42–2.01] and South‐Eastern Melbourne [OR 1.66, 95% CI 1.39–1.97]).

### Trends in diagnostic imaging over time

**Figure 3:**
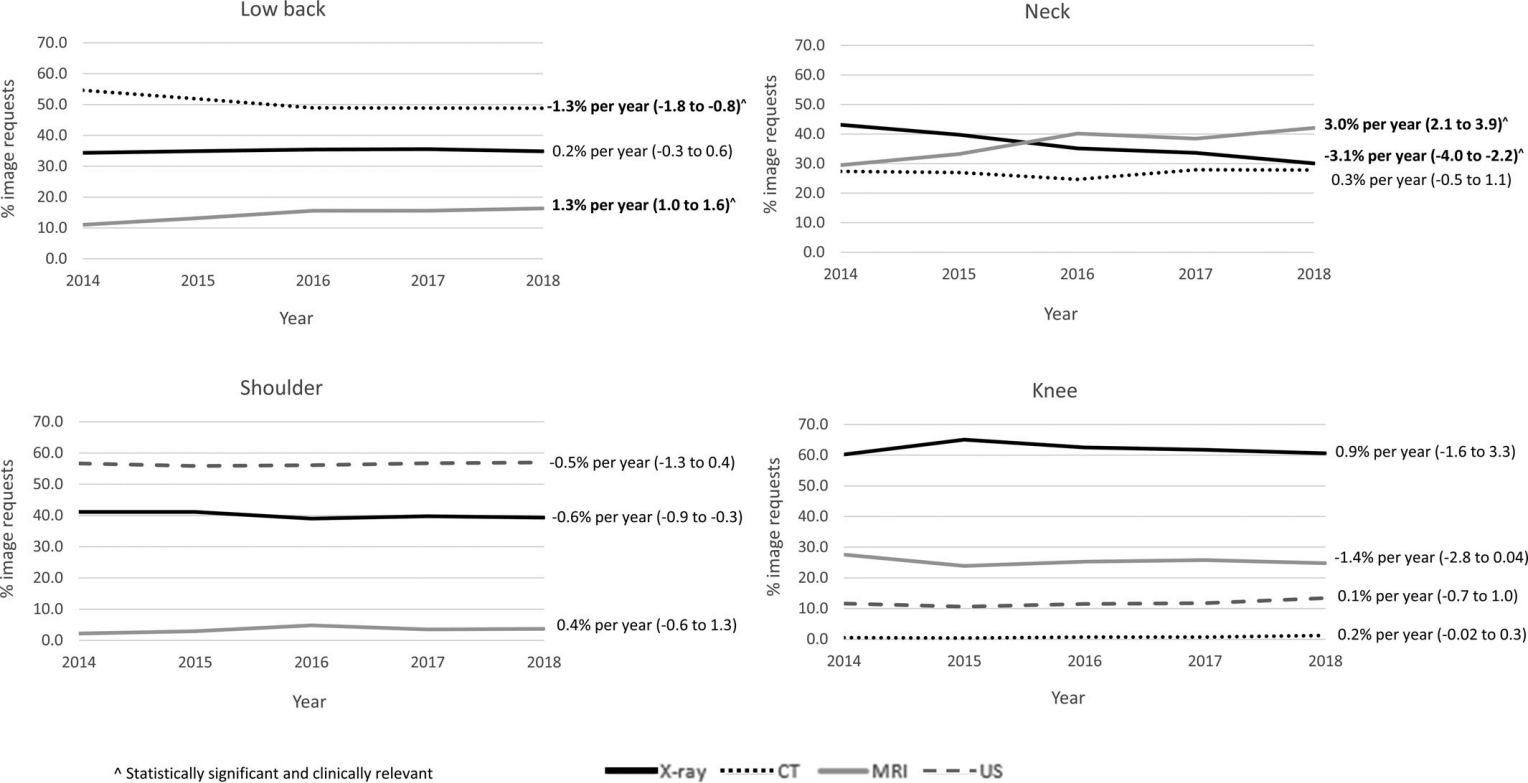
Trends in diagnostic imaging requests over time by modality and body region. CT = computed tomography; MRI = magnetic resonance imaging; US = ultrasound.

There was no appreciable change in the proportion of participants with imaging requested over the study period (Supplementary Figure 2).However, there was a change in the modalities requested for people with low back and neck conditions. There was a 1.3% (95% CI 1.0–1.6) annual increase in the proportion of requests for low back MRI and a corresponding 1.3% (95% CI 0.8–1.8) decrease in low back CT requests (Figure 3). There was a 3.0% (95% CI 2.1–3.9) annual increase in the proportion of neck MRI requests and a corresponding 3.1% (95% CI 2.2–4.0) reduction in neck x‐ray requests. There were no changes over time in the imaging modalities requested for people with shoulder or knee complaints.

### Sensitivity analyses

Sensitivity analyses including all eligible diagnostic imaging requests made during the study period (n = 94,451 images requested, 41% patients) did not appreciably change the results (Supplementary Table 1).

## DISCUSSION

Our study of routine Australian general practice based upon longitudinal consultation data indicates that GPs frequently request diagnostic imaging for people with regional musculoskeletal complaints, and this most commonly occurs at the time the diagnosis is made. Although we observed no change in overall imaging rates over the 5‐year study period, there was a trend toward more complex imaging requests in patients with neck and low back complaints. Diagnostic imaging modality varied by musculoskeletal complaint and, to a lesser extent, by patient socioeconomic status and gender and practice location. We also observed that over 1 in 10 people with a shoulder complaint had at least one image‐guided procedure.

Our findings are broadly consistent with previous studies. Previous estimates of imaging request rates by Australian GPs have varied between 33% and 43% for shoulder complaints (13, 23), 25% and 36% for knee complaints (13, 24), and 15% and 24% for patients with low back complaints (13, 25). Another study found 23% (95% CI 21–24%) of patients with a first visit for neck pain had imaging requested (25). The slightly lower rates compared with our study is likely because these studies reported point prevalence rates from a single consultation, whereas our study measured cumulative imaging request rates over the course of 1 year.

Clinical practice guidelines across all four musculoskeletal complaints and clinical care standards for low back and knee complaints (8, 9, 10) consistently recommend against routine imaging unless there are clinical features suggestive of serious pathology. Based upon the small number of people attending general practice with serious pathology, it is likely our findings are at odds with recommended practice. For example, the estimated proportion of people attending primary care with low back pain who have serious pathology ranges from 1% to 6% (26, 27, 28). Overuse of imaging for musculoskeletal complaints has also been recognized as a low‐value practice that should be questioned across several Australian and international Choosing Wisely recommendations (11, 29). Yet, our data indicate that, to date, these have had little if any impact in changing practice.

To our knowledge, this is the first study to examine the timing of GP imaging requests relative to diagnosis. We found the majority of diagnostic imaging requests occurred at or around the same time as the diagnosis with 31% occurring in the 2 weeks before diagnosis, although we cannot discount the possibility that our cohort received previous care for the same complaint or that imaging was performed only after a period of unsatisfactory improvement. Qualitative research, GP surveys, and Australian Medicare Statistics suggest both clinicians and patients continue to have misconceptions about the value of diagnostic imaging for musculoskeletal complaints (24, 30, 31, 32, 33). Shoulder MRI, knee CT, and low back MRI were the only modalities to be requested after diagnosis, suggesting perhaps that these are requested if initial management does not help or if symptoms do not subside.

Our finding that about one quarter of people who present with low back pain receive diagnostic imaging is consistent with a systematic review that found one in four patients attending primary care receive imaging (12). Our finding of a trend towards more MRI and less CT requests for low back pain over our 5‐year study period is also largely in keeping with this review, which found an increase in complex imaging from 7.4% in 1995 to 11.4% in 2015. Other studies have also reported a trend toward more complex imaging over time for low back (12, 13, 34) and neck complaints (13), although our study has now demonstrated that neck MRI requests have surpassed neck x‐ray requests. Our finding of a relative increase in lumbar spine MRI over CT requests may be partially because of a concerted effort to reduce unnecessary radiation exposure from imaging (14) even though GP‐requested lumbar spine MRI is nonrebatable in Australia. A move towards more complex imaging is not only concerning because of the heightened risk of overdiagnosis and overtreatment and greater financial cost (35), but also the carbon footprint of MRI and CT is 23 and 12 times greater than plain x‐ray, respectively (36), indicating these tests also have a significant environmental impact.

Almost 30% of our cohort with a shoulder complaint received an x‐ray request. Although plain radiographs may be worthwhile to diagnose glenohumeral joint arthritis and assess its severity, the radiographic prevalence of this condition in primary care is only 17% and mainly affects older adults (37), indicating likely overuse. Over 40% of our cohort had a shoulder ultrasound request, although the utility of diagnostic ultrasound for shoulder complaints in primary care is of questionable, if any, utility (7). Age‐related abnormalities of the rotator cuff in asymptomatic people are common and may not be of clinical relevance to a patient's complaint (3). This overuse may also partially explain the high number of patients who received an ultrasound‐guided shoulder injection. Although there is high‐quality evidence that glucocorticoid injection provides worthwhile short‐term benefit for people with rotator cuff disease (38), there is also moderate‐certainty evidence that ultrasound guidance does not improve pain or function over landmark‐guided injection (39). It also has significantly greater cost. Medicare statistics data indicate an increasing number of ultrasound‐guided injections are being performed over time, which led to it being identified as a low‐value practice, which should be questioned by the Australian Rheumatology Association (11). Other than a lack of awareness of the evidence and promotion of the procedure by vested interests, the removal of subsidized landmark‐guided intra‐articular steroid injection from the Medicare Benefits Schedule in 2009 (40) may also explain the increased use of ultrasound‐guided shoulder injection. Although meta‐analyses support the use of hydrodilatation with glucocorticoid over glucocorticoid injection alone for frozen shoulder (41), only 1.7% (n = 445) of our cohort with a shoulder complaint had a request for hydrodilatation compared with nearly 11% for injection. This may be explained by the relatively low prevalence of frozen shoulder compared with rotator cuff disease for which there is also evidence supporting the effectiveness of shoulder injection (42).

Our study has demonstrated disparities in imaging by gender, socioeconomic status, and practice location, which are consistent with known gender, socioeconomic, and geographic disparities in access to health care (43). In particular, we found patients attending practices within predominantly metropolitan PHNs were more likely to receive requests for MRI scans and ultrasound but less likely to receive x‐ray requests than those attending practices within predominantly regional and remote PHNs. This is related to limited availability of both ultrasound and MRI services and availability of experts to operate the equipment and interpret results in regional and remote areas (44). For example, less than 4% of Victorian sonographers are known to be located in our predominantly regional and remote PHN (Gippsland) (45), yet this services an area containing 8% of the Victorian population (46). It is also possible these geographic disparities are partially related to supplier‐induced demand in metropolitan areas (47).

There are many reasons why practice differs from guideline recommended care. A metasynthesis of 11 studies (n = 270) identified social influence from patients, beliefs that a scan will reassure patients, and a lack of time to discuss why a scan is not needed as the major barriers to reducing imaging for low back pain (48). Policies to address inequitable access to imaging may also inadvertently facilitate inappropriate imaging (49) as  well as fee‐for‐service models that do not remunerate for the time taken to explain why imaging is not necessary and advise on alternate management approaches (50). Successful implementation of tailored interventions to improve the appropriate use of imaging will therefore likely require a multifaceted approach targeting patients, clinicians, and health care policy.

Few interventions have been proven to reduce unnecessary imaging for musculoskeletal complaints. A Victorian mass media campaign that aimed to alter societal and clinician beliefs about low back pain performed in the late 1990s successfully improved beliefs about imaging (51), and this was sustained over time (52). Further study of ways of changing societal views about diagnostic imaging is also necessary. A metasynthesis of qualitative studies found that the general public value the information that imaging provides and also have differences in comprehension and acceptance of overuse concepts (53).

A more recent successful approach was an Australia‐wide factorial cluster trial of individualized audit and feedback targeting top requestors of 11 commonly overused musculoskeletal diagnostic imaging tests. This significantly decreased the rate of imaging requested over 6, 12, and 18 months compared with no audit and feedback (54). Further study of this relatively simple, low cost, and easily scalable intervention targeting clinicians to reduce overused diagnostic imaging tests is warranted.

We examined imaging requested by GPs in a large sample of people with regional musculoskeletal complaints that are broadly representative of the wider population (22), which is a study strength. Another strength was that we were able to capture all imaging requests irrespective of whether or not they would attract a government subsidy, whereas Medicare statistics are only able to capture tests that receive a government subsidy. Limitations of our study include that we do not know how many patients received imaging because the POLAR dataset did not include these data. We also could not determine the clinical appropriateness of the imaging requested. Our estimates of the patients receiving imaging requests are likely to be an undercount because we did not include an entire year of follow‐up for patients diagnosed in 2018. We excluded younger adults (<45 years) with knee, shoulder, and neck complaints as well. Nonaccredited, corporate‐owned general practices and those without EMRs are also likely underrepresented, and our findings may not be generalizable to them. Further, there may be some variability in our estimates of the timing of imaging requests because GPs may record a diagnosis at the first presentation or at a later visit when the diagnosis is confirmed. We also assumed that the imaging request was related to the diagnosis. Although it is possible that an imaging test could be requested for another reason, it is unlikely their frequency would be sufficient to alter our results. Similarly, it is unlikely exclusion of a small proportion of general practices because of inconsistent activity recording (11%) or uncoded tests (<5%) would have substantially altered our findings.

GPs frequently request diagnostic imaging for people with regional musculoskeletal complaints, and this most commonly occurs simultaneously with the diagnosis. It is likely a substantial proportion of requests are discordant with evidence‐based practice. Identification and testing of strategies that target patients, clinicians, and policy to improve appropriate use of imaging in people with musculoskeletal complaints is urgently needed.

## AUTHOR CONTRIBUTIONS

All authors were involved in drafting the article or revising it critically for important intellectual content, and all authors approved the final version to be submitted for publication. Dr. Haas had full access to all of the data in the study and takes responsibility for the integrity of the data and the accuracy of the data analysis.

### Study conception and design

Haas, O'Connor, Buchbinder.

### Acquisition of data

Haas, O'Connor, Buchbinder.

### Analysis and interpretation of data

Haas, Gorelik, Pearce, Mazza, Buchbinder.

## Supporting information


Disclosure Form

**Appendix S1:** Supporting Information
