# Supplementary material for: Patterns of Imaging Requests By General Practitioners for People With Musculoskeletal Complaints: An Analysis From a Primary Care Database
Source: Arthritis Care Res (Hoboken). 2023 Sep 27;77(3):402–11. doi: 10.1002/acr.25189 (PMC11848978; doi:10.1002/acr.25189)
Supplement: Supplementary file 2 — Appendix S1: Supporting Information [file ACR-77-402-s002.pdf]

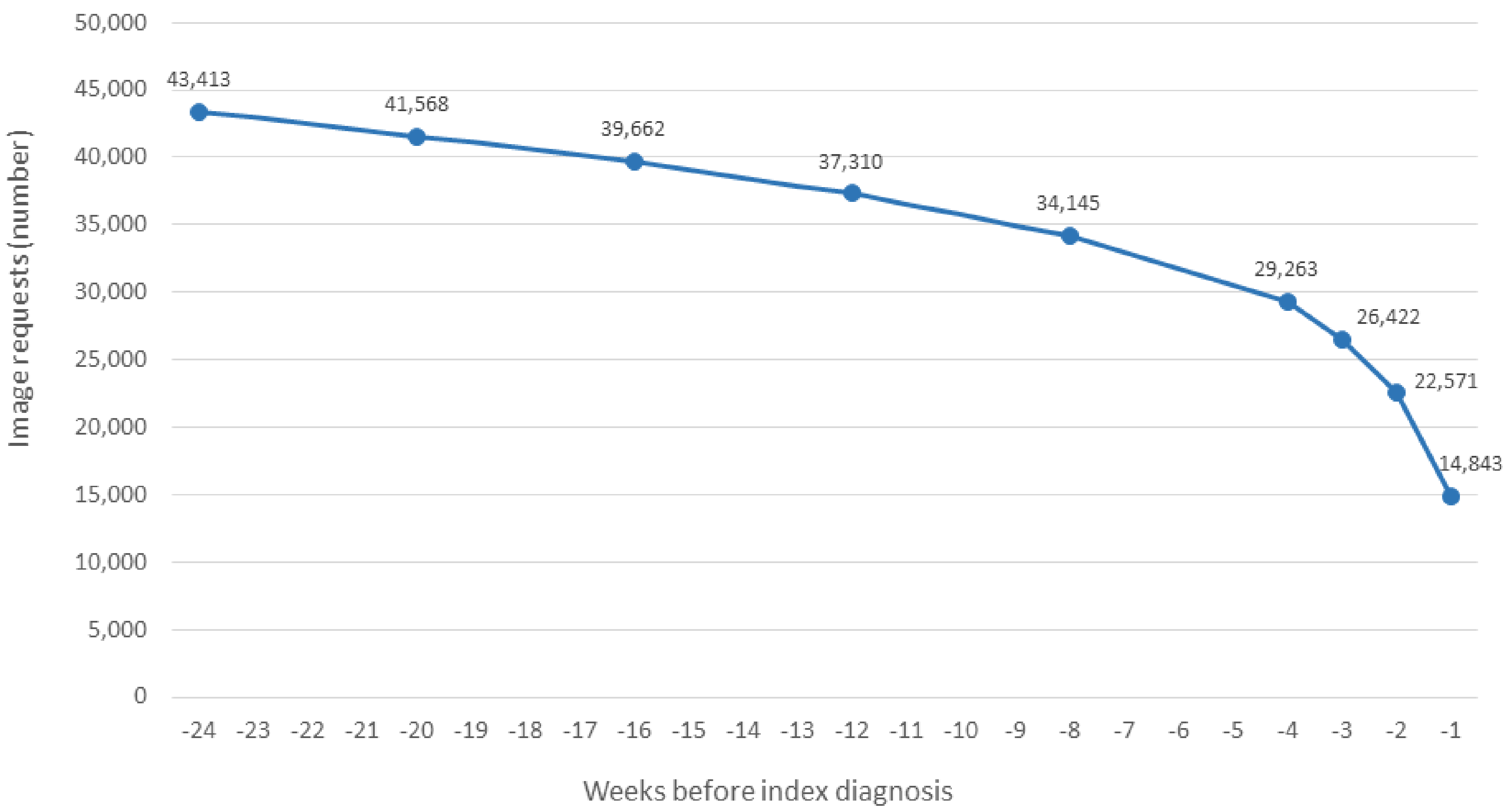

Supplementary figure 1: Number images requested during the six months before index diagnosis

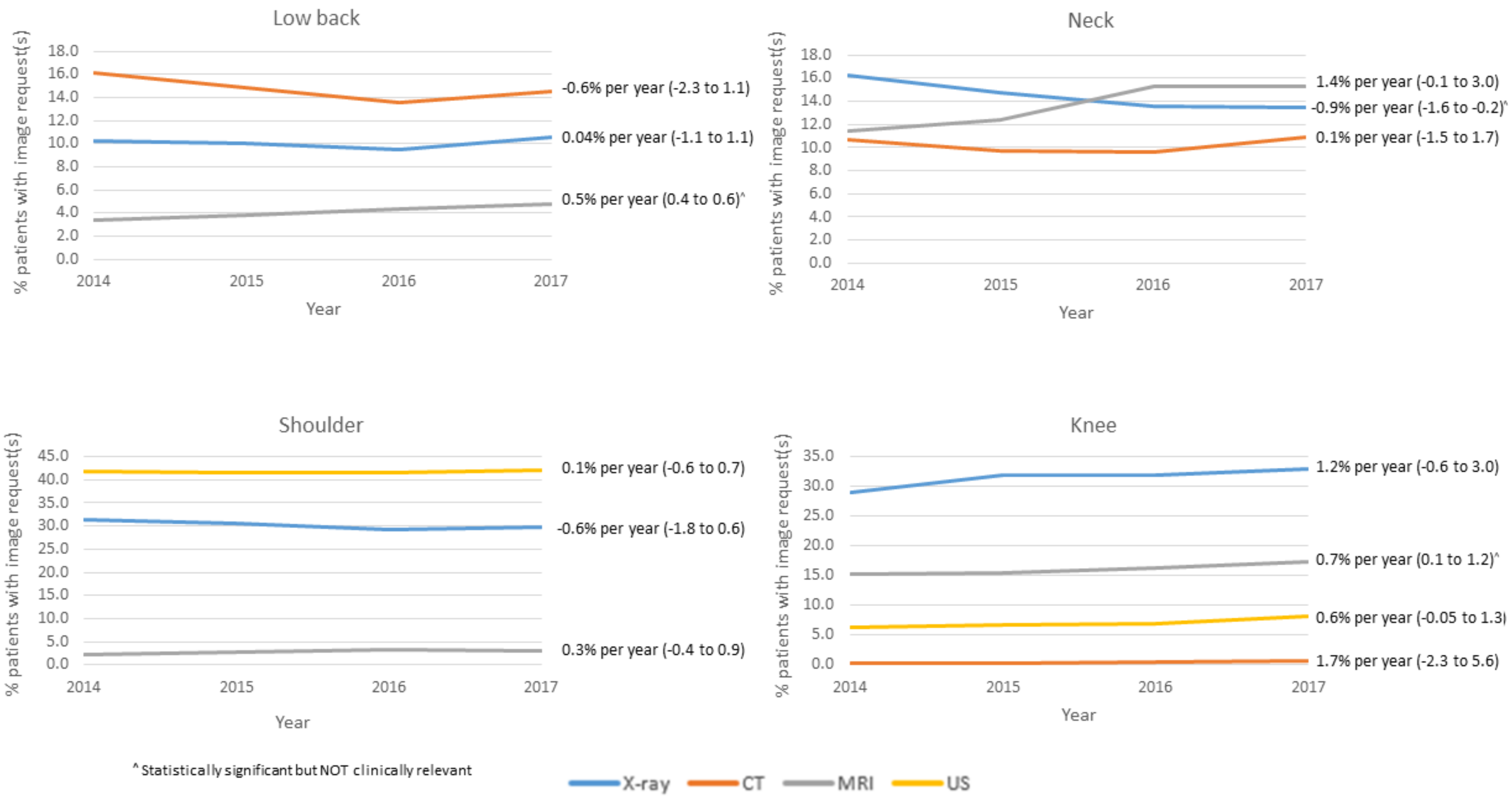

Supplementary figure 2: Trends in diagnostic imaging request rates over time by modality and body region

Participants diagnosed within the 2018 calendar year were not followed up for the full 12 months and were therefore excluded from this analysis.

Supplementary table 1: Number (%) of diagnostic and procedural imaging requests and patients (%) with imaging requests by modality and body region within entire follow-up period

	Total study cohort (133,279 patients) n (%)	Low back (76,504 patients)* n (%)	Neck (14,492 patients)* n (%)	Shoulder (26,335 patients)* n (%)	Knee (33,438 patients)* n (%)
**Patients with diagnostic imaging requests**					
**At least one request**	**50,185 (37.7)**	**22,605 (29.4)**	**5,320 (36.7)**	**13,861 (52.6)**	**16,304 (48.8)**
X-ray	30,207 (22.7)	9,005 (11.8)	2,122 (14.6)	8,608 (32.7)	12,174 (36.4)
CT scan	14,548 (10.9)	12,809 (16.7)	1,671 (11.5)	N/A	222 (0.7)
MRI scan	12,721 (9.5)	3,926 (5.1)	2,249 (15.5)	888 (3.4)	5,923 (17.7)
Ultrasound	14,332 (10.8)	N/A	N/A	11,709 (44.5)	2,852 (8.5)
**Requests for diagnostic imaging**					
**Total**	**89,166 (100)**	**27,874 (31.3)#**	**6,377 (7.2)#**	**25,906 (29.1)#**	**29,009 (32.5)#**
X-ray	41,350 (46.4)	9,761 (35.0)	2,228 (35.0)	10,459 (40.4)	18,902 (65.2)
CT scan	15,940 (17.9)	13,954 (50.1)	1,749 (27.4)	N/A	237 (0.8)
MRI scan	14,158 (15.8)	4,159 (14.9)	2,400 (37.6)	975 (3.8)	6,624 (22.8)
Ultrasound	17,718 (19.9)	N/A	N/A	14,472 (55.8)	3,246 (11.2)
**Patients with procedural imaging request^**					
**At least one request**	**4,274 (3.2)**	**370 (0.5)**	**62 (0.4)**	3,604 (13.7)	262 (0.8)
Image-guided injection	3,869 (2.9)	370 (0.5)	62 (0.4)	3,226 (12.2)	262 (0.8)
Hydrodilatation	492 (0.4)	N/A	N/A	492 (1.9)	N/A
**Requests for procedural imaging^**					
**Total**	**5,285 (100)**	**448 (8.4)#**	**73 (1.4)#**	**4,459 (84.4)#**	**305 (5.8)#**
Image-guided injection	4,710 (89.1)	448 (100)	73 (100)	3,884 (86.8)	305 (100)
Hydrodilatation	575 (10.9)	N/A	N/A	575 (13.2)	N/A

* Number of participants with a musculoskeletal condition affecting each body region sums to more than 133,279 because n=15,176 participants were diagnosed with musculoskeletal complaints affecting multiple body regions. For proportion of total radiology requests, each patient may have had requests for multiple images and/or modalities for the same body region.

Percentage of imaging requests out of those within total study cohort (n=89,166 diagnostic, n=5,285 procedural)

^Image-guided injections were intra-articular or bursal injection of glucocorticoid or it was not specified or hydrodilatation (arthrographic distension with glucocorticoid and saline, or it was not specified).
